# Effects of modified release hydrocortisone on restoration of early morning cortisol, quality of life, and fatigue in adrenal insufficiency (The CHAMPAIN study): a randomised, double-blind, double-dummy, cross-over study comparing Chronocort and Plenadren

**DOI:** 10.1016/j.eclinm.2025.103714

**Published:** 2026-01-02

**Authors:** Alessandro Prete, Verena Theiler-Schwetz, Wiebke Arlt, Jon Hazeldine, Irina-Oana Chifu, Birgit Harbeck, Catherine Napier, John D.C. Newell-Price, D. Aled Rees, Nicole Reisch, Günter K. Stalla, Helen Coope, Kerry Maltby, John Porter, Jo Quirke, Richard J. Ross

**Affiliations:** a Department of Metabolism and Systems Science, School of Medical Sciences, College of Medicine and Health, University of Birmingham, United Kingdom; b National Institute for Health and Care Research (NIHR) Birmingham Biomedical Research Centre, United Kingdom; c MRC Laboratory of Medical Sciences, London, United Kingdom; d Institute of Clinical Sciences, Imperial College London, London, United Kingdom; e Department of Inflammation and Ageing, School of Infection, Inflammation and Immunology, College of Medicine and Health, University of Birmingham, United Kingdom; f University Hospital of Würzburg, Germany; g Department of Medicine, University Hospital Hamburg, Germany; h Amedes MVZ Hamburg, Hamburg, Germany; i Newcastle University, Newcastle upon Tyne NHS Foundation Trust, United Kingdom; j University of Sheffield, Sheffield, United Kingdom; k Cardiff University, Cardiff, United Kingdom; l Medizinische Klinik und Poliklinik IV, LMU Klinikum München, Germany; m Medicover Neuroendocrinology Munich, Munich, Germany; n Neurocrine UK Ltd, Cardiff, United Kingdom

**Keywords:** Adrenal insufficiency, Hydrocortisone, Glucocorticoid, Chronocort, Plenadren

## Abstract

**Background:**

Cortisol has a circadian rhythm with an early morning rise, loss of this rhythm is associated with poor health. Our objective was to test the hypothesis that restoring the early morning cortisol rise will improve fatigue and quality of life (QoL) by comparing twice daily Chronocort with once daily Plenadren in patients with adrenal insufficiency.

**Methods:**

A randomised, double-blind, double-dummy, cross-over study with no washout in 58 patients (29 in each arm) with primary adrenal insufficiency comparing four weeks' Chronocort, 15 mg at night and 10 mg in the morning, a formulation that restores early morning cortisol levels, with four weeks’ once daily Plenadren 25 mg, which only restores daytime cortisol levels. The primary endpoint was the 07:00 h serum cortisol level and secondary endpoints measures of fatigue and QoL. The trial was registered on ClinicalTrials.gov and EU Clinical Trials Register (NCT NCT05222152; Eudract 2021-000144-21), initiated on 11th January 2022 and completed on 18th October 2023.

**Findings:**

Patients met the primary endpoint and achieved a physiological early morning serum cortisol, median 417 nmol/L on Chronocort versus 6 nmol/L on Plenadren (P < 0.0001). For secondary outcomes the majority of QoL and fatigue measures showed significant benefits for Chronocort including the disease-specific questionnaire AddiQol (P = 0.02), the fatigue questionnaire PROMIS 7b (P = 0.02), SF-36 physical component score (P = 0.01), and EQ-5D-5L (P = 0.02). The Multidimensional Assessment of Fatigue (MAF) was not significantly different between treatments; however, a pre-specified sensitivity analysis showed that in the first treatment period, Chronocort reduced the MAF Score (P = 0.008), suggesting a carry-over effect from period 1 to 2. A post hoc analysis of immune profile in a subset of 19 patients showed that those on Chronocort had an increase in circulating number or frequency of neutrophils, natural killer and natural killer T cells compared to both baseline glucocorticoid treatment and Plenadren treatment.

**Interpretation:**

Restoring the early morning cortisol levels with twice daily Chronocort 15 mg at night 10 mg in the morning improved health-related quality of life, fatigue and the immune profile compared with 25 mg daily Plenadren.

**Funding:**

Neurocrine UK Ltd.

**Research in context**

**Evidence before this study**

Adrenal Insufficiency (AI) is associated with fatigue and impaired quality of life. A recent Lancet review of Adrenal Insufficiency stated: “Reduced health-related quality of life is commonly assumed, but not proven, to be caused by the inability of standard replacement therapy to mimic the circadian and ultradian rhythmicity of cortisol”.^1^ The only blinded randomised controlled trial used a subcutaneous infusion to mimic the cortisol circadian rhythm and showed no difference in subjective health status between hydrocortisone infusion and daytime oral hydrocortisone replacement.^2^ However, this was in only 10 patients.

**Added value of this study**

We performed a Double-Blind, Double-Dummy, Two-Way Cross-Over, Randomised, Study of Efficacy, Safety and Tolerability of Modified-Release Hydrocortisones: Chronocort® Versus Plenadren®, in Adrenal Insufficiency (The CHAMPAIN study). Chronocort is a formulation that restores the early morning rise in cortisol, while Plenadren only restores daytime cortisol levels. Using this study design, we were able to test the hypothesis that restoring the physiological early morning rise in cortisol improves the subjective health status of patients with AI.

**Implications of all the available evidence**

Restoring the physiological early morning rise in cortisol improves patient reported outcomes including fatigue and quality of life.

## Introduction

A central tenet in medicine is that disruption of homeostatic mechanisms leads to disease, and effective therapy must re-establish normal physiology. Circadian rhythms have evolved in virtually all organisms to maintain homeostasis through the 24-h day/night cycle. In humans, the central clock in the suprachiasmatic nucleus controls the diurnal sleep/wake cycle, which metabolically is a fast/feed cycle. The clock maintains homeostasis by synchronising the hormones that maintain glucose homeostasis, such as insulin and the glucocorticoid cortisol. Loss of synchrony between the clock and these hormonal circadian rhythms results in loss of homeostasis as evidenced by obesity, depression, diabetes and insulin resistance in people undertaking shift work.^3^

Adrenal insufficiency (AI) is the failure of the adrenal gland to secrete the essential stress hormone cortisol and if left untreated, results in death through an adrenal crisis.^1^ AI can be either primary (adrenal), secondary (pituitary), or tertiary (hypothalamic) and treatment in all three is to replace the missing hormone cortisol (pharmaceutical name for which is hydrocortisone).^4^ Hydrocortisone treatment has been available to patients with AI since the 1950s; however, patients with AI still have increased mortality, impaired immune function with increased infection risk, impaired quality of life (QoL) and reduced work capacity compared to the healthy population, with fatigue being a common complaint.^1^^,^^5^^,^^6^ Normal endogenous cortisol secretion has a diurnal rhythm with levels rising in the early hours of the morning to peak on waking and then declining to low levels in the evening.^7^ On top of this diurnal rhythm, there is an ultradian rhythm of cortisol.^8^ Standard immediate-release hydrocortisone has a short serum half-life (∼1.5 h) such that treatment is usually given twice or thrice daily and patients wake with a very low cortisol level at the time when their physiological cortisol level should be highest. A recent Lancet review of AI stated: “Reduced health-related quality of life is commonly assumed, but not proven, to be caused by the inability of standard replacement therapy to mimic the circadian and ultradian rhythmicity of cortisol”.^1^

Modified-release formulations of hydrocortisone and subcutaneous infusions of hydrocortisone have been developed with the aim of improving cortisol replacement. In open-label studies using continuous subcutaneous infusion of hydrocortisone, there have been subjective improvements in the quality of life of AI patients.^9^ The only blinded randomised control trial, however, showed no difference in subjective health status between infusion and oral hydrocortisone.^2^ Plenadren™ (Takeda Ltd. UK), approved in Europe to treat AI, is an extended-release hydrocortisone with an outer coating layer that provides an immediate drug release and an extended-release core. Taken first thing in the morning, Plenadren provides a rapid peak in serum cortisol, followed by an extended serum profile of cortisol compared to immediate-release hydrocortisone tablets. A single morning dose of Plenadren gives similar 24 h serum cortisol exposure compared to a thrice daily regime of immediate-release hydrocortisone but both give overexposure in the morning after dosing compared to controls and Plenadren leads to lower levels in the afternoon and evening based on salivary cortisol and cortisone assessments.^10^^,^^11^ Open-label studies with Plenadren have shown an improvement in quality of life, reduction in central adiposity, and improved immune function in adult AI patients.^12^

Modified-release hydrocortisone hard capsules, development name Chronocort®, are approved in Europe to treat congenital adrenal hyperplasia (CAH) (Efmody™, Neurocrine BV. Netherlands). Chronocort differs from Plenadren in having a delayed and sustained absorption profile rather than an immediate and sustained release profile.^13^ Chronocort restores physiological cortisol concentrations by dosing at night and morning such that the night-time dose releases hydrocortisone in the early morning hours, providing a physiological pre-waking rise in cortisol levels in CAH patients^14^ and improving biochemical control of CAH patients.^15^ Prior to the CHAMPAIN study reported here, Chronocort had not been trialled in patients with causes of AI other than CAH.

The pharmacokinetic profile of Plenadren only restores the daytime cortisol levels, while Chronocort restores both the early morning rise in cortisol and daytime cortisol levels. Thus, we now have the tools to test the hypothesis that restoration of the physiological overnight rise in cortisol has more beneficial effects than daytime cortisol replacement alone. We faced challenges in study design including the fact that there are no biomarkers of disease control and patients require cortisol replacement so there could be no placebo-only arm and no washout. There is one disease-specific QoL tool, AddiQol, that includes measures of fatigue,^16^ but no fatigue specific questionnaires are validated for AI. To address this, we used a selection of health-related QoL and fatigue measures in a double-blind study and in a subset performed an analysis of the immune profile.

## Methods

### Study population

This study was a double-blind, double-dummy, two-way cross-over, randomised study conducted at eight investigational sites in 8 adult specialist endocrinology centres in UK and Germany comparing twice-daily Chronocort with once-daily Plenadren in patients with primary AI. Primary AI was defined as early morning serum pre-dose cortisol <50 nmol/L and currently treated with daily glucocorticoid as replacement therapy. Key inclusion criteria: male or female participants aged ≥18 years; participants on stable glucocorticoid treatment for ≥3 months prior to the screening visit; participants on a stable dose of fludrocortisone (if applicable) for ≥3 months prior to the screening visit. Key exclusion criteria: participants with CAH; participants with secondary or tertiary AI; past or current history of Cushing's syndrome; adrenal suppression induced by exogenous steroids; participants with insulin-treated diabetes; participants who routinely work night shifts. Full details of inclusion and exclusion criteria are given in the Supplementary Data (Supplementary File S1).

### Ethics

All participants gave written informed consent. The study was approved in the United Kingdom by the South Central–Oxford C Research Ethics Committee (Ref 21/SC/0275), approved by the Medicines and Healthcare Products Regulatory Agency. The trial was registered on ClinicalTrials.gov and EU Clinical Trials Register: Clinicaltrials.gov registration No. NCT05222152 (www.clinicaltrials.gov, registered 2 Feb 2022); EudraCT registration No. 2021-000144-21 (https://eudract.ema.europa.eu, registered 27 Aug 2021). The study was approved in Germany by the ethics committee of the Ludwig-Maximilians-University Munich (Ref 21-0986 fed) and approved by BfArM (Bundesinstitut Fur Arzneimittel und Medizinprodukte). The trial was initiated on 11th January 2022 and completed on 18th October 2023.

### Protocol

**Fig. 1:**
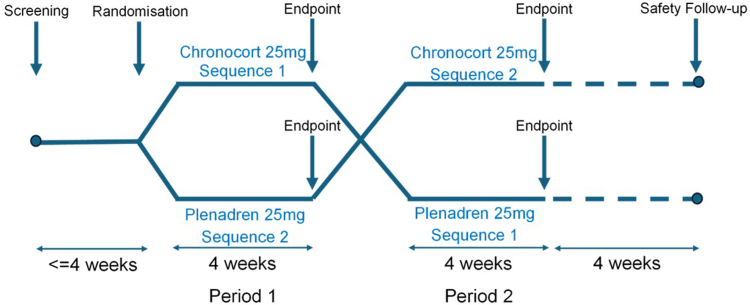
A double-blind, double-dummy, two-way cross-over, randomised study comparing twice-daily Chronocort with once-daily Plenadren in patients with primary AI. Following a screening period of up to 4 weeks, during which participants remained on their standard therapy, participants were randomised by interactive response technology (IRT) to either sequence 1 (Chronocort in period 1, followed by Plenadren in period 2) or sequence 2 (Plenadren in period 1 followed by Chronocort in period 2). Following completion of period 1, participants crossed over directly into period 2, without washout. Upon completion of period 2 participants returned to treatment under the advice of their endocrinologist, and safety follow-up continued for 4 weeks. In the crossover-designed study, efficacy endpoint variables were assessed ‘after 4-weeks of treatment’, i.e. by combining data from periods 1 and 2.

Screening period of up to 4 weeks, two 4-week cross-over treatment periods, and a 4-week follow-up period (Fig. 1). Following a screening period of up to 4 weeks, participants were randomised on 1:1 basis to blinded treatment on either Treatment Sequence I (Chronocort then Plenadren) or Treatment Sequence II (Plenadren then Chronocort) using interactive response technology (IRT). The total daily dose of Chronocort or Plenadren was 25 mg, based on international guidelines,^4^ and the Plenadren Summary of Product Characteristics (SmPC). Plenadren 25 mg was taken in the morning on waking (typically between 06:00 and 08:00 h). Chronocort 10 mg was taken in the morning on waking (typically between 06:00 and 08:00 h) and Chronocort 15 mg was taken at night just before going to bed (typically between 22:00 h and midnight). Placebo Chronocort capsules and Plenadren tablets were provided to maintain the study blind. That is, patients took in the morning either active Plenadren tablets and placebo Chronocort capsules or placebo Plenadren tablets and active Chronocort capsules and at night either placebo Chronocort capsule or active Chronocort capsule. Thus, all patients at all times were taking twice daily medication and neither physician nor patient knew which formulation they were taking as medication was prepared in packs. Participants were given stress dosing rules and emergency treatment packs of immediate release hydrocortisone. If a stress dose was taken within 48 h before any visit, then the visit was delayed until the participant had a 48-h period without use of any stress doses. All subsequent visits were then moved out accordingly. Salivary cortisone and serum cortisol samples were taken on Days 1, 28 and 56, additional salivary cortisone samples were taken on Days 21 and 49. QoL questionnaires and Visual Analogue Scales (VAS) were completed at regular intervals at the same time in the day, depending on the instrument requirements (Supplementary File S2: Patient Reported Outcome Measures Table). Other outcome measures such as sleep, and physical activity were monitored daily using a wearable device.

### Primary efficacy variable

The primary efficacy outcome variable was the 07:00 h serum cortisol level after 4 weeks of treatment.

### Key secondary variable

General fatigue using the Multidimensional Assessment of Fatigue (MAF) questionnaire.

Secondary efficacy variables:

• Physiological morning cortisol levels defined as >140 nmol/l.^4^
• Closeness of salivary cortisone levels during the day to a normal physiological profile.
• Adrenocorticotropic hormone (ACTH) control in the morning.
• Morning fatigue using the Patient-Reported Outcomes Measurement Information System (PROMIS® 7b) questionnaire within 1 h of waking.
• QoL using the EuroQol 5-level Standardised Health Questionnaire (EQ-5D-5L™).
• QoL using the Health-related Quality of Life in Addison's disease (AddiQoL) questionnaire.
• QoL using the 36-Item Short Form Health Survey (SF-36®) questionnaire.

Exploratory efficacy variables:

• Activity (Using Actigraph CentrePoint® Insight Watch).
• Sleep (Using Actigraph CentrePoint® Insight Watch).
• QoL using the Sponsor's alertness visual analogue scale (VAS).
• QoL using the General Anxiety Disorder-7 (GAD-7) scale.
• QoL using the Patient Health Questionnaire-9 (PHQ-9) scale.
• QoL using the Giessen Complaints Questionnaire-24 (GBB-24) scale (German participants only).
• Preference for treatment.

### Assays

Serum cortisol and salivary cortisone (collected by passive drool) were extracted via liquid–liquid extraction and quantified by liquid chromatography tandem mass spectrometry (LC-MS/MS). Serum cortisol: Serum samples (200 μL) were extracted by liquid–liquid extraction using charcoal stripped foetal bovine serum as surrogate matrix and cortisol-d4 as internal standard. Extracted samples (10 μL) were injected into a Kinetex XB-C18 column coupled to a Waters® Xevo™ TQ-S tandem mass spectrometer, analysed with Waters UNIFI acquisition software. Salivary cortisone: Saliva samples (200 μL) were extracted by liquid–liquid extraction and cortisone-d8 as internal standard. Extracted samples (≤10 μL) were injected into ACE 5 C18-AR column coupled to a Waters® Xevo™ TQ-S tandem mass spectrometer, analysed with Waters UNIFI acquisition software. The lower and upper limits of detection were 2.76 nmol/L to 1660 nmol/L and between run coefficient of variation ≤6.8% for serum cortisol. For salivary cortisone the lower and upper limits of detection were 2.50 nmol/L to 1670 nmol/L and between run coefficient of variation ≤3.7%. Serum cortisol values below the lower limit of quantification were imputed as half the LLOQ, and salivary cortisone values below the lower limit of quantitation (LLOQ) were imputed as equal to the LLOQ. Plasma ACTH was measured at ACM Laboratories Ltd (UK), using the Siemens Immulite 2000 assay with an analytical measuring range of 1.10 pmol/L (5 pg/mL) to 275.26 pmol/L (1250 pg/mL). Due to transition to a new assay platform and method, one participant had samples analysed using Diasorin Liaison XL assay, which had an analytical measuring range of 0.84 pmol/L (3.80 pg/mL) to 330.3 pmol/L (1500.00 pg/mL).

### Patient reported outcome measures (PROMS)

Table in Supplementary Data File 2 provides details of each measure including frequency of measurement, scale and reference to use in other disease states.

### Sample size calculation

50 evaluable participants (with an evaluable participant being a member of the Efficacy Evaluable Analysis Set [EEAS]) was considered sufficient to reject with 85% power, and at a 1-sided alpha of 2.5%, the null hypothesis that there was no difference between the effects of Chronocort and Plenadren on 07:00 h serum cortisol after 4 weeks of treatment when the true effect was that the mean 07:00 h serum cortisol after 4 weeks of Chronocort treatment was 33% higher than that after 4 weeks of Plenadren treatment.

### Statistics

Efficacy analyses were primarily based on the EEAS which required the participant to have evaluable serum cortisol data at the end of both treatment periods. The primary analysis of serum cortisol and other analyses of continuous outcome variables used a linear mixed model with participant within sequence as a random effect and treatment, period and sequence as fixed effects. Superiority of Chronocort to Plenadren was declared if the 95% confidence interval (CI) for the treatment effect was wholly above zero. Only participants with non-missing data at the timepoint of interest for each endpoint were included in the linear mixed model, no imputation of missing data was performed, n/N for each treatment arm are reported. The primary (serum cortisol) and key secondary (MAF) endpoints were controlled for multiple comparisons in a hierarchical testing scheme (i.e., test primary first, if significant, proceed to testing key secondary) and everything else was considered nominal. Unless otherwise stated, all P-values quoted are one-sided. There was a prespecified subgroup analysis of the primary efficacy outcome variable, with groups defined as: Sex (Male, Female); Age at screening, years (≥18–<30, ≥30–<50, ≥50–<70, ≥70); Baseline serum cortisol, nmol/L (≤Q1, >Q1 and ≤Q3, >Q3); Time since diagnosis of AI, years (≤Q1, >Q1 and ≤Q3, >Q3). A pre-specified sensitivity analysis was also performed to address potential carryover effects on the 8 primary and secondary outcome measures, only including Period 1 data in the analysis. This was performed using an analysis of covariance (ANCOVA) model including treatment as main effect and log-baseline serum cortisol as continuous covariate, P-values were considered nominal. Whether or not a participant had achieved a physiological morning cortisol level was compared between treatments using a Mainland-Gart test. The salivary cortisone profiles were compared to those of a healthy population.^17^ Immunology data were analysed by fitting a mixed model, after log-transformation, the results are nominal and no adjustment was made for multiple biomarkers.

### Post hoc immunology sub-study

A post-hoc immune profile sub-study was conducted at the University of Birmingham in samples from 19 participants from 2 sites who consented to collection of additional research samples. Complete blood differential counts and measurement of neutrophil reactivity and granularity were performed using a haematology analyser (Sysmex XN-1000, Sysmex, Germany). Peripheral blood mononuclear cells (PBMCs) were isolated from heparin anti-coagulated blood samples within 4 h of collection, and Natural Killer (NK) cells were isolated from PBMCs by negative selection using magnetic-assisted cell sorting (Miltenyi Biotec, Gladbach, Germany). Analyses of NK cell function included NK cell cytotoxicity assay and CD107a degranulation assay, which are described in detail in the Supplementary File S3.

### Role of the funding source

This work was initiated and supported by Neurocrine U.K. Ltd. (previously Diurnal Ltd.). No authors were paid to write the manuscript and authors were not precluded from accessing data in the study and accept responsibility to submit for publication.

## Results

### Participants

**Fig. 2:**
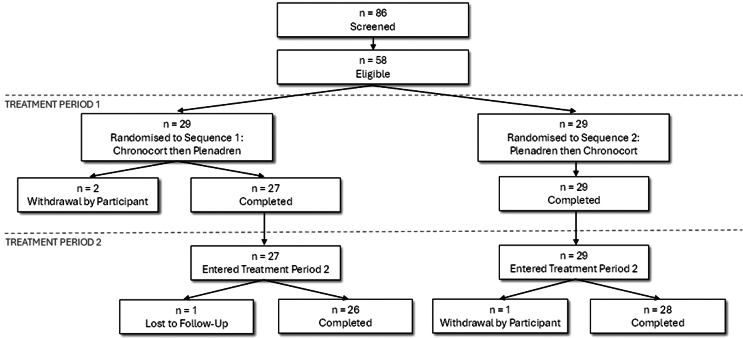
Consort diagram disposition of patients. The Safety Analysis Set (SAF) comprised all participants randomised into the study who received any dose of study drug (N = 58). The Full Analysis Set (FAS) comprised all participants who had serum cortisol assessment at the end of treatment period 1 (N = 55). The Efficacy Analysis Evaluable Set (EEAS) comprised participants from the FAS without a major significant protocol deviation, who had evaluable serum cortisol at the end of both treatment periods (N = 49). Reasons for exclusions from the EEAS: Sequence 1, n = 6 (major protocol deviation n = 3, missing serum cortisol result n = 3); Sequence 2, n = 3 (major protocol deviation n = 2, missing serum cortisol result n = 1).

At baseline, the demographic characteristics, hormones and PROM data were similar in the two treatment sequences (Table 1). The median (range) daily dose of hydrocortisone at baseline was 20 mg (15–60) with no significant difference between the two sequences. All patients were on immediate release hydrocortisone at baseline except for one patient who was on prednisolone 3 mg twice daily at baseline which was converted to 30 mg hydrocortisone equivalent. Eighty-six participants were screened, 58 enrolled, and 29 treated with Chronocort followed by Plenadren and 29 with Plenadren followed by Chronocort. Fifty-four (93.1%) participants completed the study and 4 withdrew (two in Period 1, one in Period 2, and one lost to follow-up). All 58 randomised participants were included in the safety analysis set (SAF), 55 (94.8%) were included in the Full Analysis Set (FAS), and 49 (84.5%) were included in the Efficacy Evaluable Analysis Set (EEAS); reasons for exclusion from the EEAS are detailed in Fig. 2. Median treatment compliance across both periods was 100% for both Chronocort and Plenadren and for each of the placebo treatments.

**Table 1:** Baseline demographics, hormone levels and PROM measures of the 58 enrolled study participants.

Variable	Statistic	Sequence 1 Chronocort/Plenadren	Sequence 2 Plenadren/Chronocort	Overall
Demographics	n	29	29	58
Age (years)	Mean (SD)	49.4 (14.9)	49.3 (14.6)	49.4 (14.6)
Male	n (%)	9 (31.0)	7 (24.1)	16 (27.6)
Female	n (%)	20 (69.0)	22 (75.9)	42 (72.4)
Time from diagnosis (years)	Mean (SD)	11.5 (11.0)	13.6 (11.2)	12.5 (11.1)
Height (cm)	Mean (SD)	169 (10)	170 (11)	169 (10)
Weight (kg)	Mean (SD)	74.3 (23.4)	80.0 (19.3)	77.1 (21.4)
BMI (kg/m^2^)	Mean (SD)	25.7 (6.4)	28.0 (7.1)	26.8 (6.8)
Hormones & PROM	n	20–23	24–26	
Serum Cortisol (nmol/L)^a^	Median (1st, 3rd quartile)	12.8 (1.4, 34.8)	3.2 (1.4, 9.2)	5.4 (1.4, 15.6)
ACTH (pmol/L)^a^	Median (1st, 3rd quartile)	181.3 (169.0, 275.0)	161.0 (28.4, 275.0)	173.6 (66.5, 275.0)
MAF Global Fatigue Index	Mean (SD)	26.0 (10.0)	25.1 (9.9)	25.5 (9.8)
PROMIS 7b	Mean (SD)	50.5 (12.0)	51.0 (12.5)	50.8 (12.1)
EQ-5D-SL	Mean (SD)	0.89 (0.14)	0.86 (0.13)	0.87 (0.14)
EQ-VAS	Mean (SD)	74.8 (15.5)	69.5 (20.0)	71.9 (18.1)
AddiQol	Mean (SD)	83.6 (10.5)	81.0 (13.2)	82.1 (12.0)
SF-36 PCS	Mean (SD)	47.5 (7.7)	45.8 (10.8)	46.6 (9.4)
SF-36 MCS	Mean (SD)	47.7 (9.5)	49.3 (7.5)	48.6 (8.4)
Alertness VAS	Mean (SD)	65.4 (26.5)	69.8 (25.1)	67.8 (25.5)
GAD-7	Mean (SD)	4.15 (3.41)	4.96 (5.03)	4.59 (4.34)
PHQ-9	Mean (SD)	5.85 (4.36)	7.04 (4.89)	6.50 (4.64)
GBB-24^b^	Mean (SD)	23.2 (12.5)	22.3 (14.8)	22.7 (13.4)

a Baseline hormone levels at 07:00.

b Only 19 participants as this was a Germany-only measure.

### Waking serum cortisol and plasma ACTH

**Fig. 3:**
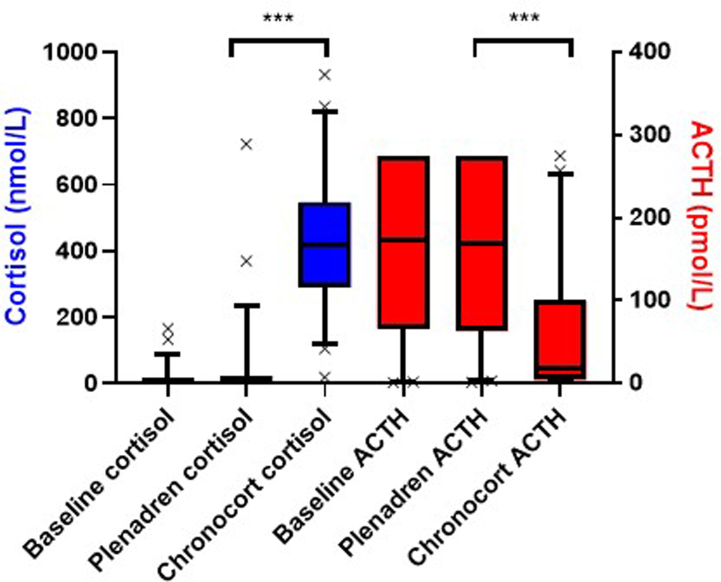
Waking serum cortisol and plasma ACTH on Chronocort and Plenadren. Data are shown as boxplots, with boxes representing median and interquartile range, and whiskers representing 5th to 95th centile. Participants included in serum cortisol analysis: Baseline n = 49; Chronocort n = 49; Plenadren n = 49. Participants included in ACTH analysis: Baseline n = 47; Chronocort n = 48; Plenadren n = 45. The majority of participants on Plenadren had a plasma ACTH at the upper limit of quantification (275 pmol/L) and only one patient had a sample diluted, which showed an ACTH of 750 pmol/L. ∗∗∗ = P < 0.0001.

**Fig. 4:**
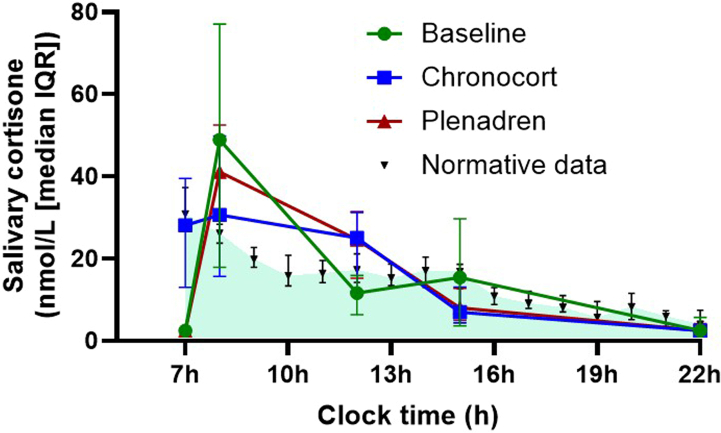
Daytime salivary cortisone profiles on immediate-release hydrocortisone, Plenadren and Chronocort. Data are shown as median and IQR. The shaded area represents normative data from 14 healthy adults with median and IQR.^17^

At baseline the median (IQR) cortisol and ACTH was 5.41 (1.38–15.9) nmol/L and 174 (67–275) pmol/L, respectively (Fig. 3). The primary efficacy outcome variable was the 07:00 h serum cortisol level after 4 weeks treatment: the median serum cortisol was 417.0 nmol/L following Chronocort and 6.04 nmol/L following Plenadren (P < 0.0001). Ninety-two percent of patients on Chronocort had a morning cortisol >140 nmol/L and only 4% of those on Plenadren (P < 0.0001). At the end of 4 weeks of treatment, median 07:00 h plasma ACTH was 19.0 pmol/L following Chronocort treatment and 169.6 pmol/L following Plenadren treatment (P < 0.0001). The majority of patients on Plenadren had a plasma ACTH reported at the upper limit of quantification (275 pmol/L); however, in one patient the sample was diluted and measured to be 750 pmol/L. For serum cortisol and salivary cortisone the 07:00 samples on Plenadren and at baseline were very low and ∼25% were below the LLOQ. Both sex subgroups, all age subgroups, all baseline serum cortisol subgroups, and all time since diagnosis of AI subgroups showed a similar response to the primary analysis. The daytime salivary cortisone profiles (Fig. 4) in patients on Chronocort confirmed that waking salivary cortisone levels were similar to a previously published healthy population,^17^ whereas waking salivary cortisone levels in Plenadren treated participants were low. The salivary cortisone peak was higher after dosing on Plenadren than Chronocort but during the rest of the day, levels were similar.

### Secondary outcomes

#### Fatigue

The key secondary efficacy outcome variable was the MAF weekly fatigue score (see table in Supplementary File S2 for details of each measure including frequency of measurement, scale and reference to use in other disease states). After 4 weeks, the LS mean difference of −2.39 between the Chronocort and Plenadren treatments was not statistically significant (P = 0.1013) (Table 2). However, a pre-specified sensitivity analysis using only Period 1 data showed both arms improved from baseline and the LS mean difference of −6.86 between the two treatments, was statistically significant in favour of Chronocort (P = 0.0082). Daily morning fatigue, measured using the PROMIS 7b questionnaire within 1 h of waking, showed a LS mean difference of −2.591 between the two treatments after 4 weeks (P = 0.0242), indicating less morning fatigue was experienced whilst on Chronocort treatment compared to Plenadren. This superiority of Chronocort in reducing morning fatigue was confirmed in the pre-specified sensitivity analysis of Period 1 data only (P = 0.0129).

**Table 2:** Summary of secondary outcomes: For 4-week data, linear mixed effects model was used for all patient reported outcome measures, using the patient reported outcome measure after 4 weeks of treatment in each study period as response and including treatment, period and sequence as fixed effects, participants were nested within sequence as random effect and a variance components covariance matrix.

Measurement	Chronocort		Plenadren		Statistics	Comment
	Mean (SD)	n/N	Mean (SD)	n/N		
MAF Global Fatigue Index (GFI)	17 (10)	42/49	20 (10)	40/49	LS Mean Diff −2.39 P = 0.101	Higher score more fatigue
MAF GFI Sensitivity period 1	15 (9)	21/23	22 (11)	19/26	LS Mean diff -6.86 P = 0.008	
PROMIS 7b	43 (9)	44/49	45 (9)	42/49	LS Mean Diff −2.6 P = 0.024	Lower score reduced fatigue
PROMIS 7b Sensitivity period 1	40 (7)	22/23	46 (12)	19/26	LS Mean Diff -6.27 P = 0.013	
EQ-5D-SL	0.887 (0.2)	43/49	0.818 (0.2)	41/49	LS Mean Diff 0.061 P = 0.021	Higher value better health
EQ-5D-5L Sensitivity period 1	0.93 (0.1)	21/23	0.79 (0.2)	20/26	LS Mean Diff 0.13 P = 0.003	
EQ-VAS	81 (15)	43/49	77 (20)	41/49	LS Mean Diff 3.2 P = 0.071	Higher value better health
EQ-VAS Sensitivity Period 1	85 (10)	21/23	71 (23)	20/26	LS Mean Diff 9.8 P = 0.023	
AddiQoL	89 (13)	43/49	85 (15)	41/49	LS Mean Diff 3.21 P = 0.023	Higher score better QoL
AddiQoL sensitivity period 1	91 (9)	21/23	82 (17)	20/26	LS Mean Diff 6.3 P = 0.018	
SF-36 Physical Component Score (PCS)	48.3 (10.2)	43/49	44.3 (10.7)	41/49	LS Mean Diff 3.2 P = 0.010	Higher score better health
PCS sensitivity period 1	49.4 (6.3)	21/23	41.0 (12.5)	20/26	LS Mean Diff 6.6 P = 0.001	
SF-36 Mental Component Score (MCS)	53.1 (7.3)	43/49	53.3 (8.6)	41/49	LS Mean Diff −0.6 P = 0.700	Higher score better health
MCS sensitivity period 1	55.4 (6.2)	21/23	53.4 (11.0)	20/26	LS Mean Diff 3.0 P = 0.120	
Treatment Preference VAS period 1	84 (24)	21/23	64 (31)	23/26	LS Mean Diff 19.8 P = 0.012	Higher score prefer treatment
Treatment Preference VAS period 2	51 (31)	23/23	43 (34)	21/26	LS Mean Diff 8.3 P = 0.198	

LS means and their differences and 95% CI were obtained from the model. Prespecified period 1 sensitivity analyses, an analysis of covariance (ANCOVA) model was applied for primary and secondary study outcomes, using data from the 4th week of treatment period 1 as response, and including treatment and baseline scores as covariates.

#### Health-related quality of life

The **EQ-5D-5L** utility score showed a LS mean difference of 0.061 between the two treatments after 4 weeks (P = 0.0212), indicating better overall health outcome was experienced whilst on Chronocort treatment compared to Plenadren (Table 2), with similar results on analysis of Period 1 data only (LS mean difference 0.13, P = 0.0026) and Chronocort score improved by 0.04 compared to baseline. At the end of 4 weeks of treatment, the mean EQ-VAS score showed a LS mean difference of 3.20 mm between the two treatments, which was not statistically significant (P = 0.0714). However, the pre-specified sensitivity analysis using Period 1 data only was statistically significant (P = 0.0233), indicating better overall health outcomes were experienced whilst on Chronocort treatment compared to Plenadren. The **AddiQoL** results showed a LS mean difference of 3.21 between the two treatments, which was statistically significant (P = 0.0236), indicating a better QoL following Chronocort treatment. Similar results were seen in the pre-specified sensitivity analysis on Period 1 data only (P = 0.0175). The **SF-36** analysis showed statistically significant differences between Chronocort and Plenadren treatment for the domains of general health perceptions (P = 0.0180) and physical component score (P = 0.0106) after 4 weeks of treatment, with the differences showing an improvement after Chronocort treatment compared to after Plenadren treatment. Similar results were seen in the pre-specified sensitivity analysis using Period 1 data only, where physical functioning, role limitations due to physical health, bodily pain and vitality were significantly improved after Chronocort.

### Exploratory outcomes

#### Activity and sleep

The wearable device showed no difference in any measure of activity or sleep between the two treatments.

#### PROMS

The **GAD-7 scale** showed no difference between the two treatments (LS mean difference −0.54, P = 0.0868) (Table 2). The **PHQ-9 scale** showed a LS mean difference of −1.11 between the two treatments, which was statistically significant (P = 0.0169), indicating better health outcomes following Chronocort treatment. The **GBB-24 scale** (conducted in the 19 German participants only) showed no difference between the two treatments (LS mean difference −1.04, P = 0.1835). The **Participant preference for assigned treatment** during Period 1 relative to pre-study treatment showed a median VAS score of 95 mm following Chronocort treatment and 65 mm following Plenadren treatment. The LS mean difference of 19.8 mm between the 2 treatments was statistically significant (P = 0.012) in favour of Chronocort (Table 2).

#### Post hoc immunology sub-study

**Fig. 5:**
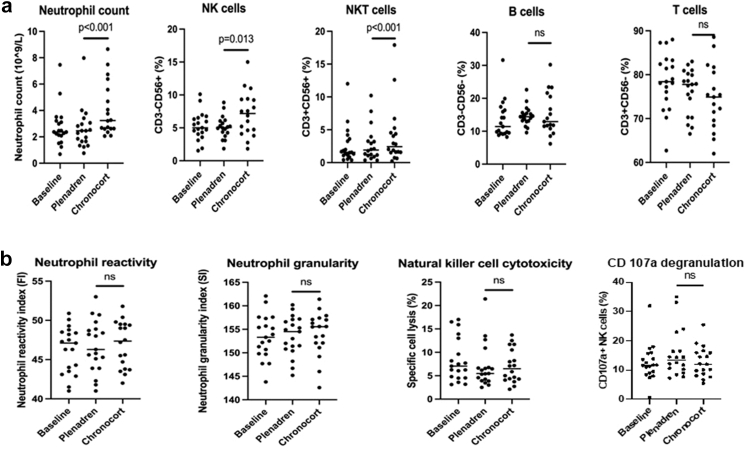
Immune analysis in 19 patients with primary adrenal insufficiency at baseline, on Plenadren, and on Chronocort at the end of the crossover. a: Immune cell counts and frequencies. b: Analysis of immune cell function. Abbreviations: NK = natural killer; NKT = natural killer T; ns = non-significant; Data were log transformed for performing mixed model analyses, but untransformed values are shown.

19 patients with primary AI were included in the immune function analyses: 12 (71%) female; mean (SD) age 53.6 (14.1) yrs (Table 3). All were on immediate-release hydrocortisone pre-randomisation, median dose 25 mg (range 20–30). Ten patients were randomised to Plenadren in Treatment Period 1 and nine to Chronocort. There was a significant increase in the neutrophil count after Chronocort compared to Plenadren treatment in the crossover study (Fig. 5a). No significant differences were observed in the neutrophil reactivity index or neutrophil granularity index (Fig. 5b). There was a significant increase in NK and NKT cell frequencies after Chronocort treatment, while no significant changes from baseline were observed with Plenadren (Fig. 5a). Neither Chronocort nor Plenadren treatment significantly affected the number of B and T cells. There was no difference in natural killer cell cytotoxicity (NKCC) or CD107a degranulation between the two treatments (Fig. 5b). A sub-analysis of immune parameters at the end of Treatment Period 1, included in the study to rule out a carry-over effect, showed similar trends (Supplementary File S3).

**Table 3:** Immune cell counts and immune function analyses in 19 patients with primary adrenal insufficiency at the end of the crossover.

	Baseline median (IQR)	Plenadren median (IQR)	Chronocort median (IQR)	P-value		
				PLN vs. BL	CHC vs. BL	PLN vs. CHC
Neutrophil count (10^9^/L)	2.37 (2.09–3.21)	2.46 (1.65–3.06)	3.24 (2.62–5.78)	0.828	0.004	<0.001
Neutrophil reactivity index (FI)	47.1 (43.5–48.8)	46.3 (43.9–48.9)	47.35 (43.78–49.5)	0.490	0.097	0.362
Neutrophil granularity index (SI)	153.3 (149.8–157.6)	154.5 (150.8–157.1)	155.6 (151.9–157.2)	0.868	0.247	0.291
NK cells (%)	5 (4.06–6.53)	5.045 (4.14–6.07)	7.18 (4.50–9.34)	0.792	0.007	0.013
NKT cells (%)	1.55 (1.09–3.61)	1.9 (1.03–3.43)	2.42 (1.56–4.72)	0.548	0.001	<0.001
B cells (%)	19.9 (9.51–78.1)	17.75 (12.7–75.03)	22.95 (13.73–71.5)	0.654	0.535	0.893
T cells (%)	71.9 (11.4–81.8)	72.45 (14.9–80.05)	63.65 (14.4–80.38)	0.272	0.099	0.581
NKCC (%)	7.06 (4.53–13)	5.47 (3.92–6.77)	6.48 (4.25–10.8)	0.464	0.599	0.999
CD107a degranulation (%)	11.66 (8.94–15.63)	13.39 (9.89–18.06)	12.03 (8.49–16.04)	0.349	0.884	0.218

Abbreviations: IQR = interquartile range, RI = reactivity index, FI = fluorescence intensity, SI = scatter intensity, NK = natural killer, NKT = natural killer T, NKCC = natural killer cytotoxicity, PLN = Plenadren, CHC = Chronocort. Data were log transformed for performing mixed model analyses, but untransformed values are shown. Samples were only collected from participants at 2 of the UK sites.

### Safety

The use of stress dosing was similar during the two treatment periods (19 participants [32.8%] had 87 occurrences of stress dosing during Chronocort treatment and 20 participants [35.7%] had 81 occurrences of stress dosing during Plenadren treatment). A total of 317 treatment emergent adverse events (TEAEs) were reported by 51 participants (87.9%), with more experiencing TEAEs with Plenadren (47/56, 83.9%) compared to Chronocort (43/58, 74.1%). The most common TEAE by preferred term was fatigue, reported by twice as many participants during Plenadren (44.6%) compared to Chronocort (20.7%). On-treatment serious adverse events (SAEs) were reported for 4 participants (6.9%), with 5 events in 3 participants (5.2%) occurring during Chronocort treatment and 1 event in 1 participant occurring during Plenadren treatment. None of the SAEs had a fatal outcome. All SAEs were associated with acute AI; 3 in combination with an infection (including influenza and Covid-19), 1 SAE of acute AI was not associated with an infection and was attributed to the participant not complying with stress-dosing rules. None of the SAEs were considered by the investigator or sponsor as related to the study treatment. No participant discontinued treatment or left the study due to a TEAE.

## Discussion

This is the first blinded interventional study comparing two modified-release oral hydrocortisone replacement therapies. It demonstrated that replacing the early morning rise in cortisol reduces morning fatigue and improves QoL and immune profile in patients with primary AI. Waking morning cortisol levels were found to vary in healthy individuals, with mean (95% CI) peak waking cortisol being 414 (320–524) nmol/L.^7^ A morning cortisol level <140 nmol/L is considered to reflect relative AI,^4^ and we used a morning cortisol <50 nmol/L to define primary AI. Following treatment, the median morning serum cortisol was 417 nmol/L on Chronocort vs 6 nmol/L on Plenadren and 92% of patients on Chronocort had a waking cortisol >140 nmol/L compared to only 4% of those on Plenadren. Thus, during the study period, the majority of patients on Chronocort woke with physiological cortisol levels whereas patients on Plenadren woke with levels defined as AI. Waking plasma ACTH levels were very elevated in patients on Plenadren and much reduced on Chronocort, providing further evidence of physiological replacement with Chronocort. Salivary cortisone levels were measured during the day and compared to physiological profiles from a published healthy population.^17^ Salivary cortisone was chosen as it better reflects serum cortisol levels,^18^ is not interfered with by oral hydrocortisone and can be measured when serum cortisol levels are low.^19^ The daytime salivary cortisone profiles in patients on Chronocort confirmed the waking salivary cortisone levels were similar to a previously published healthy population, whereas on Plenadren waking salivary cortisone levels were low. The salivary cortisone peak was higher after dosing on Plenadren than Chronocort but during the rest of the day levels were similar.

Monitoring cortisol replacement is challenging as there is no accepted biomarker. Generally, physicians follow published guidelines, and prescribe a daily dose of 15–25 mg hydrocortisone.^20^ We chose to use a daily dose of 25 mg/day as we did not want our study to be complicated by the effects of potential under-replacement. It is unlikely that the dose influenced the QoL as previous studies have suggested that increasing the daily dose of hydrocortisone does not improve the QoL in patients with AI.^21^ We chose Plenadren as our comparator because it has previously been shown to provide equivalent cortisol exposure over 24 h to thrice daily hydrocortisone,^10^ and open-label studies with Plenadren have shown an improvement in metabolic markers and quality of life.^12^^,^^22^ It was approved by the EMA as replacement therapy under orphan designation, evidence that the EMA consider Plenadren has significant clinical benefit over existing treatments. Plenadren leads to lower cortisol levels in the afternoon and evening than standard therapy,^10^^,^^11^ which has been suggested to be of metabolic benefit.^23^ Our study did not compare Chronocort to standard therapy in the blinded component of the study; however, for both Plenadren and Chronocort there were improvements in patient reported outcome measures after switching from baseline, on immediate release hydrocortisone, to either Plenadren or Chronocort.

The only other double-blind study of hydrocortisone replacement in patients with primary AI used a continuous subcutaneous infusion in 10 patients and found no impact on QoL^2^; however, the patient number was small, and the patients had a good QoL at baseline. We chose to use the disease-specific questionnaire AddiQoL and a selection of generic questionnaires in our study, and compliance with PROM completion was very good (82–90% for PROM measures used for secondary endpoints) despite the number of questionnaires. Overall, there was a consensus between all the questionnaires, showing that Chronocort treatment was associated with improved QoL. For AddiQoL, patients on Chronocort showed significantly higher values than Plenadren and a 7-point improvement from baseline. In the previously reported study of Plenadren, the AddiQoL score increased by 1 point at 3 months and 4 points at 12 months when switching from hydrocortisone tablets to Plenadren.^22^ For EQ-5D-5L, the utility score was greater on Chronocort treatment than with Plenadren. SF-36 is one of the most widely used generic QoL tools, with a higher score representing better health. The SF-36 showed statistically significant better QoL for Chronocort vs Plenadren for both general health perceptions and physical component score. The previous open label study of Plenadren vs thrice daily immediate-release hydrocortisone showed no significant difference in SF-36 measures.^10^

For fatigue, the PROMIS 7b is a generic tool that measures daily fatigue with lower scores representing less fatigue. In this study, we used it within 1 h of waking such that the question: "Since you woke up today how often did you feel tired?", would relate to morning fatigue. The PROMIS 7b was significantly lower on Chronocort vs Plenadren and the score fell from a mean of 51 at baseline on immediate release hydrocortisone to 43 after 4 weeks on Chronocort. In cancer studies, a reduction of 2–3 in the PROMIS 7b score is considered clinically significant,^24^ so a fall of 8 in AI patients taking Chronocort reflects a clinically significant improvement in fatigue. This was supported by the difference between treatments in adverse event reports, where reports of fatigue were twice as common on Plenadren compared to Chronocort: 45% vs 21% of patients. There was no other difference in adverse event reporting between the two groups, but it was interesting to note the high level of stress dosing in approximately a third of patients. The Multidimensional Assessment of Fatigue (MAF) global fatigue index was originally developed in rheumatoid arthritis with the intent to measure fatigue. It measures fatigue experienced in the past week so may not be as sensitive to the predominantly morning fatigue seen in adrenal insufficiency. A lower MAF score is associated with reduced fatigue and, though lower on Chronocort, was not significantly different, although the sensitivity analysis in the first period showed that the MAF score was significantly lower for the patients on Chronocort vs Plenadren. This suggests a period effect which might be more likely to be noted on a scale measuring symptoms over the preceding week, rather than the more immediate effects measured by the PROMIS 7b. The baseline MAF score was relatively low at 25 compared to that for patients with rheumatoid arthritis. On Chronocort in period one the MAF score fell from 26 to 15, a fall of 11 points, which is considered clinically significant especially as the score was low to start with.^25^

In a subset of participants, post hoc sub-study showed that four weeks of treatment with Chronocort led to an improved immune profile in terms of cell number but not function. This is notable, as observational studies have shown a high risk of infections, greater antimicrobial prescribing, and infection-related mortality in patients with primary AI.^5^^,^^26^^,^^27^ The reasons behind this increased infection risk are likely multifactorial including genetic factors,^28^^,^^29^ as well as unphysiological cortisol replacement.^5^ Two cross-sectional studies showed impaired NK cell function in autoimmune AI, classic congenital adrenal hyperplasia, and following bilateral adrenalectomy compared to healthy controls.^6^^,^^30^ A randomised controlled trial where patients with primary and secondary AI were switched from standard glucocorticoid treatment to Plenadren showed an increase in NK cells at 3 and 6 months, which was associated with lower rates of infections.^12^ We observed that Chronocort led to an increase in neutrophil, NK and NKT cell counts compared to Plenadren after 4 weeks: these are effector cells of the innate immune system that are critical in the direct elimination of viral, bacterial, and fungal infections. Importantly, the basal activation status of neutrophils, as measured by reactivity and granularity intensity, was comparable across treatment groups, meaning increased numbers were not associated with systemic activation, in the absence of pathogenic challenge, that could be detrimental to the host. Although there was no increase in NKCC with Chronocort treatment, the fact that the treatment did lead to an increase in NK frequency could suggest that overall cytotoxicity would be greater at a site of infection.

In this study, sleep and activity were measured as an exploratory analysis using a validated wearable device, but results demonstrated no difference between Chronocort or Plenadren. Sleep disruption has been reported as a cause of poor QoL in patients with AI,^31^ and nighttime administration of steroids in CAH is reported to cause sleep disruption.^32^ Chronocort is given last thing at night but, because of its delayed release, cortisol levels only start to rise in the early morning hours as occurs physiologically, and it is reassuring that there was no sleep disruption. One might expect that reduced fatigue would translate into greater morning activity, but this was not seen when assessed with wearables. However, a confounding factor might be that the study was performed during the Covid-19 pandemic when the healthy population took less exercise, and people with AI were practising strict social-distancing.

The safety profiles were similar for Chronocort and Plenadren, as expected for patients with AI, with no new safety signals identified. Fatigue was more commonly reported as an adverse event in patients whilst on Plenadren; otherwise, there was no difference in adverse event reporting between the two groups. Approximately a third of patients in each group reported stress dosing during the study, which seems quite high and probably reflects that patients received education about stress dosing and this was a monitored study. There were five instances of acute adrenal insufficiency but three of these were precipitated by a viral infection including influenza and Covid-19.

Plenadren is a once daily medication taken first thing in the morning and Chronocort a twice daily medication taken first thing in the morning and last thing at night. Owing to the double blind, double-dummy design during which all participants followed a twice daily treatment regimen, it is not possible to assess the impact of once-versus twice-daily dosing with active on adherence or other patient outcomes in this study. Standard immediate-release hydrocortisone gives active hydrocortisone twice or thrice daily, usually first thing in the morning, around midday, and then around 5 pm, Plenadren is a dual release, giving immediate then sustained release of hydrocortisone, and Chronocort is delayed and sustained release of hydrocortisone. The overall daily dose is split over the 24 h for the different treatments. Our results do show that QoL improved from baseline immediate-release hydrocortisone to treatment with either once daily active Plenadren or twice daily active Chronocort. Adherence is challenging to assess in the real world and there is an assumption that once daily treatment is better than taking medication through the day as required by standard hydrocortisone replacement. Adherence taking Chronocort first thing in the morning and last thing at night has not been compared to once daily medication; however, in the 4-year open label study of Chronocort in congenital adrenal hyperplasia, adherence was high at 98.9% based on study pharmacist's assessment of packs returned,^33^ suggesting that compliance is good when taking a toothbrush regimen such as Chronocort.

The limitations of this study were its short duration and cross-over design with no washout. However, it is not possible to wash out hydrocortisone as this was the treatment in both interventional arms and patients with AI cannot stop hydrocortisone replacement. Four weeks is a relatively short period for a QoL study; however, as hydrocortisone has a short half-life and this was the first blinded study of oral therapy we wanted to test if there were changes on switching therapy especially on fatigue which may change early in treatment. There was evidence of a carryover effect in period 2 for MAF which measures fatigue over the last week; however, the sensitivity analysis in period 1, which is essentially a randomised parallel arm study, confirmed MAF was significant despite the reduced power. This was an intense study being double-blind double dummy and with multiple questionnaires, but compliance was good and the discontinuation rate was low.

In conclusion, this study confirmed that replacing the early morning rise in cortisol improved the wellbeing and QoL of patients with AI.

## Contributors

Conceptualisation: all authors contributed equally. Data curation: AP, HC, KM, JP, JQ, & RJR. Formal analysis: AP, HC, KM, JP, JQ, & RJR. Project administration: KM, & JQ. Writing–original draft: RJR. Writing–review & editing: all authors contributed equally. All authors read and approved the final version of this manuscript. AP, HC, JQ, AP, DAR, & RJR had direct access to and verified the underlying data and RJR, AP, and AR are from the academic team.

## Data sharing statement

The study protocol is available at www.clinicaltrials.gov and additional analyses are available in the Supplementary Appendices. Additional information including statistical analysis plan will be supplied on request to the corresponding author.

## Declaration of interests

**The authors have the following conflicts of interest to declare in relation to this work:** A.P., D.A.R, R.J.R., and J.P. are consultants to, and H.C., K.M., J.Q., are employees, of Neurocrine U.K. Ltd. A.P. has received grant funding from Diurnal Ltd to support sample analysis, received honoraria and travel support from Diurnal Ltd to present at meetings and is a Trustee of the Addison's Disease Self-help Group. D.A.R. has received funding from Neurocrine U.K. Ltd to the institution to undertake the clinical trial, personal for attending a Neurocrine advisory board and received payment for making presentations at meetings from Neurocrine U.K. Ltd and FrostPharma AB.
