# Corrigendum to “Effects of modified release hydrocortisone on restoration of early morning cortisol, quality of life, and fatigue in adrenal insufficiency (The CHAMPAIN study): a randomised, double-blind, double-dummy, cross-over study comparing Chronocort and Plenadren”[eClinical Medicine 91(2026); 103714]

**DOI:** 10.1016/j.eclinm.2026.103787

**Published:** 2026-02-12

**Authors:** Alessandro Prete, Verena Theiler-Schwetz, Wiebke Arlt, Jon Hazeldine, Irina-Oana Chifu, Birgit Harbeck, Catherine Napier, John D.C. Newell-Price, D. Aled Rees, Nicole Reisch, Günter K. Stalla, Helen Coope, Kerry Maltby, John Porter, Jo Quirke, Richard J. Ross

**Affiliations:** a Department of Metabolism and Systems Science, School of Medical Sciences, College of Medicine and Health, University of Birmingham, United Kingdom; b National Institute for Health and Care Research (NIHR) Birmingham Biomedical Research Centre, United Kingdom; c Division of Endocrinology and Diabetology, Department of Internal Medicine, Medical University of Graz, Graz, Austria; d MRC Laboratory of Medical Sciences, London, United Kingdom; e Institute of Clinical Sciences, Imperial College London, London, United Kingdom; f Department of Inflammation and Ageing, School of Infection, Inflammation and Immunology, College of Medicine and Health, University of Birmingham, United Kingdom; g University Hospital of Würzburg, Germany; h Department of Medicine, University Hospital Hamburg, Germany; i Amedes MVZ Hamburg, Hamburg, Germany; j Newcastle University, Newcastle upon Tyne NHS Foundation Trust, United Kingdom; k University of Sheffield, Sheffield, United Kingdom; l Cardiff University, Cardiff, United Kingdom; m Medizinische Klinik und Poliklinik IV, LMU Klinikum München, Germany; n Medicover Neuroendocrinology Munich, Munich, Germany; o Neurocrine UK Ltd, Cardiff, United Kingdom

This corrigendum corrected an error found in the original article that had been published in volume 91 (2026). The authors identified that an affiliation was missing for Verena Theiler-Schwetz. Verena Theiler-Schwetz should have had the following affiliations:

- Department of Metabolism and Systems Science, School of Medical Sciences, College of Medicine and Health, University of Birmingham, United Kingdom and
- Division of Endocrinology and Diabetology, Department of Internal Medicine, Medical University of Graz, Graz, Austria

The authors would like to apologise for any inconvenience caused.

