# Supplementary material for: Effects of modified release hydrocortisone on restoration of early morning cortisol, quality of life, and fatigue in adrenal insufficiency (The CHAMPAIN study): a randomised, double-blind, double-dummy, cross-over study comparing Chronocort and Plenadren
Source: eClinicalMedicine. 2026 Jan 2;91:103714. doi: 10.1016/j.eclinm.2025.103714 (PMC12805350; doi:10.1016/j.eclinm.2025.103714)
Supplement: Supplementary File 1 [file mmc1.docx]

**Supplementary Material**

**Replacing the circadian rhythm of cortisol improves quality of life, fatigue and immune function in patients with primary adrenal insufficiency**

**Inclusion and Exclusion Criteria**

Inclusion Criteria

1. Male and female participants aged ≥18 years.
2. Participants with known (documented) primary AI, defined as early morning pre‑dose cortisol <50 nmol/L and currently treated with glucocorticoid as replacement therapy. Primary AI includes any cause of acquired or congenital primary adrenal failure including autoimmune Addison’s disease and bilateral adrenalectomy (except when performed for Cushing’s syndrome).
3. Participants on stable glucocorticoid treatment for ≥3 months prior to the Screening Visit.
4. Participants on a stable dose of fludrocortisone (if applicable) for ≥3 months prior to the Screening Visit.
5. Male participants must agree to use contraception during the Screening, Treatment, and Follow-up Periods and refrain from donating sperm during these periods and for 7 days after the last dose of study treatment.
6. A female participant is eligible to participate if she is not pregnant, not breastfeeding, and for whom at least one of the following conditions applies: not a woman of childbearing potential (WOCBP) or a WOCBP with a negative urine pregnancy test at entry into the study who agrees to follow the contraceptive guidance during the Screening, Treatment and Follow-up Periods and for 7 days after the last dose of study treatment.
7. Capable of giving signed informed consent, which includes compliance with the requirements and restrictions listed in the ICF and in the protocol.

Exclusion Criteria

1. Participants with CAH.
2. Participants with secondary and tertiary AI.
3. Past or current history of Cushing’s syndrome.
4. Adrenal suppression and/or AI induced by exogenous steroids.
5. Drug-induced AI.
6. Clinical or biochemical evidence of hepatic disease: elevated liver function tests (alanine aminotransferase [ALT] or aspartate aminotransferase [AST] >3 times the upper limit of normal [ULN]).
7. Clinical or biochemical evidence of renal disease: serum creatinine level of >221 µmol/L (2.5 mg/dL) or calculated creatinine clearance of <25 mL/min.
8. History of malignant brain tumours or traumatic brain injury.
9. History of malignancy within the last 5 years or treated basal cell carcinoma within the past year.
10. Participants who have type 1 diabetes or type 2 diabetes receiving regular insulin.
11. Participants with type 2 diabetes whose screening HbA1c exceeds 9%.
12. Participants who have elective surgical procedures scheduled during the study.
13. Participants with significant medical or psychiatric conditions that in the opinion of the Investigator would preclude participation in the study.
14. Participants who have had bariatric surgery within the past 6 months and participants who plan to undertake a major weight loss and/or exercise program during the same time period as anticipated study involvement.
15. Restless legs syndrome/Willis-Ekbom disease.
16. Participants who have increased gastrointestinal motility e.g., chronic diarrhoea, that may be at risk of impaired cortisol exposure. There are no data in patients with confirmed slow gastric emptying or decreased motility disease/disorder so the clinical response should be monitored in patients with these conditions.
17. Participants anticipating regular prophylactic use of additional steroids e.g., for strenuous exercise.
18. Participants with co-morbidities requiring daily administration of a medication (or consumption of any material) that interferes with the metabolism of glucocorticoids.
19. Participants on regular daily inhaled, topical, nasal, or oral steroids for any indication other than AI.
20. Participants who have received intra-articular steroid injections within 1 year prior to the Screening Visit or for whom such injections are planned during the study.
21. Participants who are receiving <10 mg hydrocortisone dose at the Screening Visit or the hydrocortisone dose equivalent.
22. Participants taking sleeping medication.
23. Participants treated at screening with either Chronocort or Plenadren.
24. Participation in another clinical study of an investigational or licensed drug or device within 12 weeks or 5 half-lives prior to the Screening Visit or at any time during study participation.
25. Active alcohol or drug abuse within 1 year prior to the Screening Visit.
26. Participants who routinely work night shifts and do not sleep during the usual night‑time hours.
27. Participants who intend to travel and cross a time zone of greater than ±3 hours within 1 week of the scheduled visit dates.
28. Participants unable to comply with the requirements of the protocol in the opinion of the Investigator.
29. Participants with a known hypersensitivity to any of the components of the Chronocort capsules, the Plenadren tablets, the Chronocort placebo, or the Plenadren placebo

**Trial Registration Information**

**EudraCT number:** 2021-000144-21

**Clinicaltrials.gov number:** NCT05222152
