# Supplementary material for: Effects of modified release hydrocortisone on restoration of early morning cortisol, quality of life, and fatigue in adrenal insufficiency (The CHAMPAIN study): a randomised, double-blind, double-dummy, cross-over study comparing Chronocort and Plenadren
Source: eClinicalMedicine. 2026 Jan 2;91:103714. doi: 10.1016/j.eclinm.2025.103714 (PMC12805350; doi:10.1016/j.eclinm.2025.103714)
Supplement: Supplementary File 2 [file mmc2.docx]

**Patient Reported Outcome Measures Table**

| Scale | Objective | Description of scoring range | Scoring Period | Scale Administration Schedule | Timepoints used in statistical analyses | MCID* and disease state from which it was generated |
| --- | --- | --- | --- | --- | --- | --- |
| MAF (Multidimensional Assessment of Fatigue) | To measure self-reported fatigue in adults with chronic illness | The Global Fatigue Index score ranges between 1 to 50. Lower scores indicatee less fatigue. | Over the past week | Completed at Baseline and then weekly thereafter within 1hr of waking until the end of TP2. | **Secondary Endpoint:** End of TP1, End of TP2 **Sensitivity Analysis:** Baseline, End of TP1 | 11-18 point reduction (Rheumatoid Arthiritis) (2) |
| PROMIS-7b (Patient-Reported Outcomes Measurement Information System - 7-Item Short Form) | To evaluate daily fatigue | Scores range from 31 to 81. Lower scores indicate less fatigue. | Over the past 24-hours | Completed at Baseline and then daily thereafter within 1hr of waking until the end of TP2. | **Secondary Endpoint:** The averaged daily measurements collected during the 4th week of TP1 and TP2 **Sensitivity Analysis:** Baseline, 4th week of TP1 | 2-3 point reduction (Oncology) (3) |
| EQ-5D-5L (European Quality of Life 5-dimension 5-level Scale) | To assess health outcome from a wide variety of interventions on a common scale, for purposes of evaluation, allocation and monitoring | Health Index Scores range from less than 0 (representing health states worse than death) to 1 (representing perfect health). The exact range varies slightly depending on the specific population^3^  EQ VAS scores - self-rated health on a scale from 0 (‘the worst health you can imagine’) to 100 (‘the best health you can imagine’). | Health state at the time of completion | Completed at Baseline and then weekly thereafter within 2hrs of waking until the end of TP2. | **Secondary Endpoint:** End of TP1, End of TP2 **Sensitivity Analysis:** Baseline, End of TP1 | The average (range) MCID value of the EQ-5D-5L was 0.071 (0.052–0.098) (Coronary Heart Disease) (4) |
| AddiQoL (Addison's Disease-specific Quality of Life) | To quantify altered well-being and treatment effects in Addison’s disease | Total scores range from 30 to 120. Higher scores indicate better QoL. | Quality of Life at the time of completion | Completed at Baseline and at the end of TP1 and TP2 within 2hrs of waking. | **Secondary Endpoint:** End of TP1, End of TP2 **Sensitivity Analysis:** Baseline, End of TP1 | **Not reported (5)** |
| SF-36 (Short Form (36) Health Survey) | To measure generic health concepts relevant across age, disease, and treatment groups | Scores range from 0 to 100. Higher scores indicate better overall health | Over the past week | Completed at Baseline and then weekly thereafter within 2hrs of waking until the end of TP2. | **Secondary Endpoint:** End of TP1, End of TP2 **Sensitivity Analysis:** Baseline, End of TP1 | General (non–disease-specific) MCID: 2 points in PCS; 3 points in MCS; 2 to 4 points for individual dimensions  SF-36v2 User’s manual |
| PHQ-9 (Patient Health Questionnaire-9 | To measure depression severity and to diagnose depressive disorders | Scores range from 0-27. Lower scores indicate better general health outcomes | Over the last 2-weeks | Completed at Baseline and then every 2-weeks thereafter within 2hrs of waking until the end of TP2. | **Exploratory Endpoint:** End of TP1, End of TP2 | MCID is estimated as 2 points in total score (Depression) (6) |
| GAD-7 (General Anxiety Disorder-7 Scale) | To identify probable cases of Generalized Anxiety Disorder (GAD) and assess symptom severity in GAD | Scores range from 0 to 22. Lower scores equating to less anxiety | Over the last 2-weeks | Completed at Baseline and then every 2-weeks thereafter within 2hrs of waking until the end of TP2. | **Exploratory Endpoint:** End of TP1, End of TP2 | MCID is estimated as 4 points in total score (Chronic Depression) (7) |
| GBB-24 (Giessen Subjective Complaints List) | To assess the frequency and intensity of physical complaints | Scores range from 0-96. Lower scores indicate better health outcomes. | Over the past week | Completed at Baseline and at the end of TP1 and TP2 within 2hrs of waking. | **Exploratory Endpoint:** End of TP1, End of TP2 | **Not reported** |
| Alertness VAS | To measure alertness | Scores range from 0 to 100. A score of 0 indicates 'Brain Fog' and a score of 100 being 'Fully-Alert’. | Alertness at the time of completion | Completed at Baseline and then daily thereafter within 1hr of waking until the end of TP2. | **Exploratory Endpoint:** The averaged daily measurements collected during the 4th week of TP1 and TP2 | NA |
| Treatment Preference VAS | To measure preference for treatment | Scores range from 0 to 100. A higher score indicates preference for the most recent treatment received. | NA | Completed at the end of TP1 and the end of TP2 | **Exploratory Endpoint:** End of TP1, End of TP2 | NA |

***MCID = Minimal Clinically Important Difference**

**References**

1. Godoy-Ramirez K, Franck K, Gaines H. A novel method for the simultaneous assessment of natural killer cell conjugate formation and cytotoxicity at the single-cell level by multi-parameter flow cytometry. *J Immunol Methods*. 2000;239(1-2):35-44.

2. Pouchot J, Kherani RB, Brant R, Lacaille D, Lehman AJ, Ensworth S, Kopec J, Esdaile JM, Liang MH. Determination of the minimal clinically important difference for seven fatigue measures in rheumatoid arthritis. *Journal of clinical epidemiology*. 2008;61(7):705-713.

3. Nordin A, Taft C, Lundgren-Nilsson A, Dencker A. Minimal important differences for fatigue patient reported outcome measures-a systematic review. *BMC Med Res Methodol*. 2016;16:62.

4. Zheng Y, Dou L, Fu Q, Li S. Responsiveness and minimal clinically important difference of EQ-5D-5L in patients with coronary heart disease after percutaneous coronary intervention: A longitudinal study. *Front Cardiovasc Med*. 2023;10:1074969.

5. Oksnes M, Bensing S, Hulting AL, Kampe O, Hackemann A, Meyer G, Badenhoop K, Betterle C, Parolo A, Giordano R, Falorni A, Papierska L, Jeske W, Kasperlik-Zaluska AA, Chatterjee VK, Husebye ES, Lovas K. Quality of life in European patients with Addison's disease: validity of the disease-specific questionnaire AddiQoL. *J Clin Endocrinol Metab*. 2012;97(2):568-576.

6. Kounali D, Button KS, Lewis G, Gilbody S, Kessler D, Araya R, Duffy L, Lanham P, Peters TJ, Wiles N, Lewis G. How much change is enough? Evidence from a longitudinal study on depression in UK primary care. *Psychol Med*. 2022;52(10):1875-1882.

7. Toussaint A, Husing P, Gumz A, Wingenfeld K, Harter M, Schramm E, Lowe B. Sensitivity to change and minimal clinically important difference of the 7-item Generalized Anxiety Disorder Questionnaire (GAD-7). *J Affect Disord*. 2020;265:395-401.
