# Supplementary material for: Effects of modified release hydrocortisone on restoration of early morning cortisol, quality of life, and fatigue in adrenal insufficiency (The CHAMPAIN study): a randomised, double-blind, double-dummy, cross-over study comparing Chronocort and Plenadren
Source: eClinicalMedicine. 2026 Jan 2;91:103714. doi: 10.1016/j.eclinm.2025.103714 (PMC12805350; doi:10.1016/j.eclinm.2025.103714)

**Supplementary Material**

**Replacing the circadian rhythm of cortisol improves quality of life, fatigue and immune function in patients with primary adrenal insufficiency**

**1. Immunology Sub-study**

Natural killer cell cytotoxicity assay (NKCC): NKCC was assessed using a modified version of the previously described protocol (1). Briefly, K562 target cells were cultured either alone or with NK cells at an effector:target (E:T) cell ratio of 10:1 for 4 h at 37°C in a humidified 5% CO_2_ atmosphere. After incubation, cells were pelleted and resuspended in PBS/1%BSA containing 0.3 µg anti-CD56 PE (Miltenyi Biotec). Following a 20-minute incubation on ice, samples were washed in PBS and stained for 5 min with 125 nM of the dead cell stain Sytox blue (Life Technologies) prior to flow cytometric analysis. To measure NKCC, the number of lysed K562 target cells (defined as sytox blue positive) in a total population of 2,000 was recorded. From here, the percentage of specific cell lysis was calculated as: (TL-SL/2000) × 100, where TL is the number of lysed target cells in NK-K562 co-culture samples and SL is the number of K562 cells that underwent lysis when cultured with media alone.

CD107a degranulation assay: 2x10^5^ PBMCs, resuspended in RPMI-1640 media supplemented with 10% (volume/volume (v/v)) heat-inactivated (HI) foetal calf serum (FCS; Sera Laboratories International, Sussex, UK), 2 mM L-glutamine, 100 U/ml penicillin and 100 µg/ml streptomycin (Sigma-Aldrich, Dorset UK), hereafter referred to as complete media (CM), were cultured for 1 hour at 37^o^C with 100 µl CM or K562 cells (2x10^6^/ml in CM) in the presence of 1.25 µg/ml of a FITC-conjugated mouse anti-human CD107a monoclonal antibody (eBiosciences, Hatfield, Ireland, UK; Clone eBioH4A3) or its concentration matched isotype control (mouse IgG1-FITC; Dako). Post-incubation, 6 µg/ml of monensin was added to all samples, which were incubated for an additional 5 hours at 37^o^C. Post-incubation, samples were stained with 1 µg/ml of a PcB-conjugated mouse anti-human CD3 (BD Biosciences, Oxford, UK) and 0.3 µg/ml of a PE-conjugated mouse anti-human CD56 antibody (Miltenyi BioTec). After a 20-minute incubation on ice, samples were washed once in PBS and CD107a expression was measured by flow cytometry, where the percentage of CD107a positive NK cells was recorded alongside the mean fluorescence intensity value. CD107a expression was measured by gating on CD3^-^ CD56^+^ cells within the lymphocyte pool. CD107a expression on NK cells cultured with CM alone was considered background and the value was subtracted from each test sample.


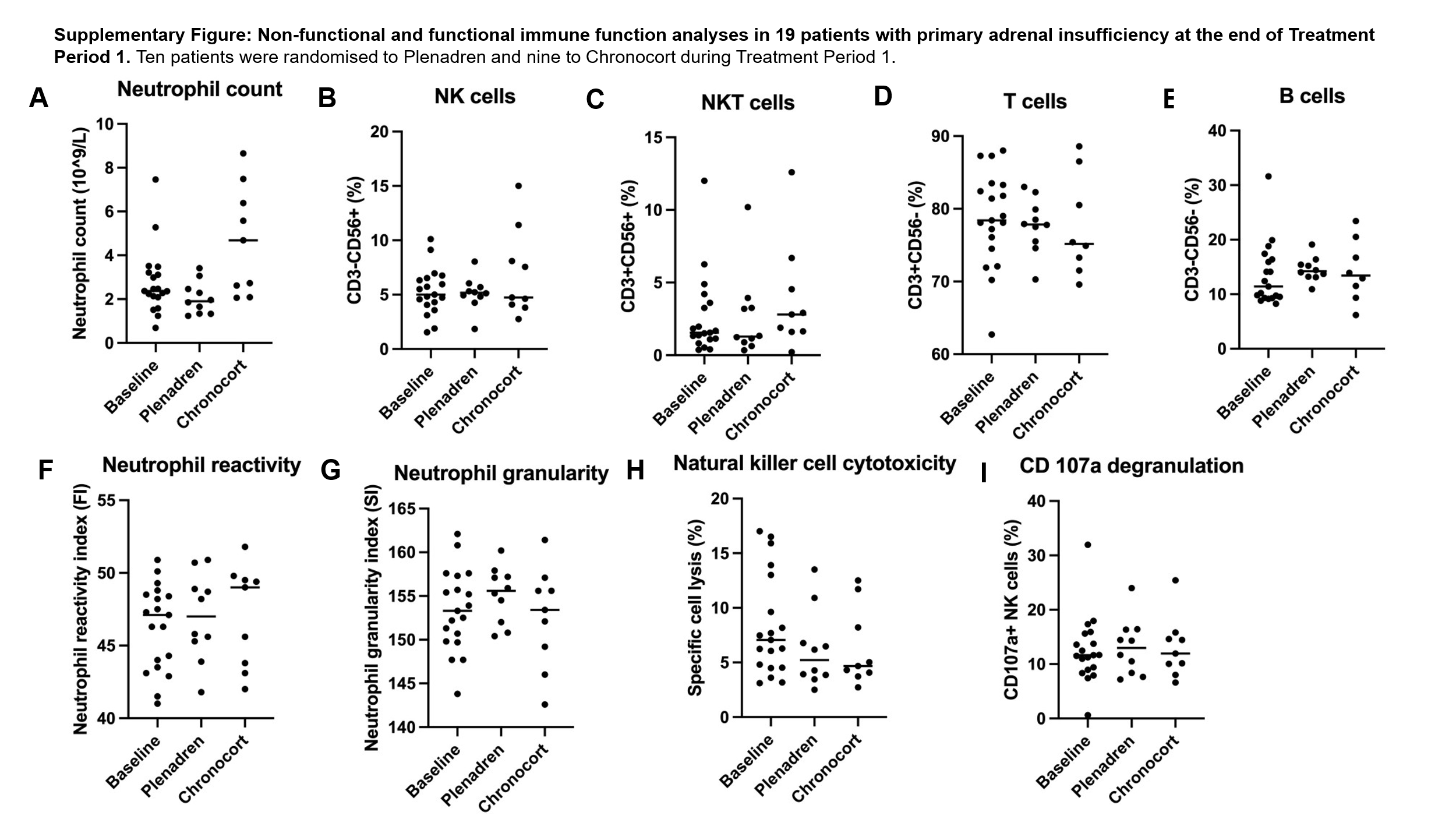

Supplement: Supplementary File 3 [file mmc3.docx]
